# Molecular Landscape for Malignant Transformation in Diffuse Astrocytoma

**DOI:** 10.1055/s-0041-1731069

**Published:** 2021-06-22

**Authors:** Thara Tunthanathip, Surasak Sangkhathat, Kanet Kanjanapradit

**Affiliations:** 1 Department of Surgery, Division of Neurosurgery, Faculty of Medicine, Prince of Songkla University, Hat Yai, Songkhla, Thailand; 2 Department of Surgery, Faculty of Medicine, Prince of Songkla University, Hat Yai, Songkhla, Thailand; 3 Department of Biomedical Sciences, Faculty of Medicine, Prince of Songkla University, Hat Yai, Songkhla, Thailand; 4 Department of Pathology, Faculty of Medicine, Prince of Songkla University, Hat Yai, Songkhla, Thailand

**Keywords:** malignant transformation, diffuse astrocytoma, whole genome sequencing

## Abstract

**Background** Malignant transformation (MT) of low-grade gliomas changes dramatically the natural history to poor prognosis. Currently, factors associated with MT of gliomas have been inconclusive, in particular, diffuse astrocytoma (DA).

**Objective** The present study aimed to explore the molecular abnormalities related to MT in the same patients with different MT stages.

**Methods** Twelve specimens from five DA patients with MT were genotyped using next-generation sequencing (NGS) to identify somatic variants in different stages of MT. We used cross-tabulated categorical biological variables and compared the mean of continuous variables to assess for association with MT.

**Results** Ten samples succussed to perform NGS from one male and four females, with ages ranging from 28 to 58 years. The extent of resection was commonly a partial resection following postoperative temozolomide with radiotherapy in 25% of cases. For molecular findings, poly-T-nucleotide insertion in isocitrate dehydrogenase 1 (IDH1) was significantly related to MT as a dose–response relationship (Mann–Whitney's *U* test, *p* = 0.02). Also, mutations of *KMT2C* and *GGT1* were frequently found in the present cohort, but those did not significantly differ between the two groups using Fisher's exact test.

**Conclusion** In summary, we identified a novel relationship between poly-T insertion polymorphisms that established the pathogenesis of MT in DA. A further study should be performed to confirm the molecular alteration with more patients.

## Introduction

Malignant transformation (MT) of low-grade gliomas (LGGs), which is the progression of benign tumor cells converting to malignancy, was found in 19.5 to 21%. 1 2 3 4 The 10-year cumulative incidence of MT was 3.8% and the median time of MT was 5.1 years from a study of Broniscer et al, 1 while other prior studies reported MT rates in LGGs in 19.5%. 2 Additionally, the MT of these benign tumors changed the natural history of the disease and also accelerated to a poor prognosis. 4 5 6

Currently, the pathogenesis of MT is still being debated. Murphy et al studied 599 patients with LGG and found the incidence of MT was 21%. From the results of prior studies, risk factors associated with MR were older age, male gender, multiple tumors, chemotherapy alone, and the extent of resection were potential predictors of MT. 4 However, radiation exposure was reported as a significant factor associated with MT in the study of Sakarunchai et al. 2 Furthermore, molecular alterations had an impact on MT in various previous studies. TP53 overexpression, deletions of *RB1* , *CDKN2A* , and PTEN pathway abnormalities were related to MT. 1 4 Based on the 2016 World Health Organization (WHO) central nervous system (CNS) tumor classification, Jakola et al revealed that *IDH* -mutant 1p/19q-intact and *IDH* -wild-type LGG were significantly associated with MT. 6

As MT in LGG, especially diffuse astrocytoma (DA), is not a common event, the transformation processes need time and long-term follow-up. Hence, a lack of evidence of MT in the specific group of DA comprised benign astrocyte cells directly turning to malignant cells from the literature review. The heterogeneity in various types of LGG such as DA, oligodendroglioma might interfere with the result of genetic alterations related to MT. 2 3 Moreover, a comparison of genetic alterations in the same patient has been rarely reported. Hence, we conducted a molecular study of DA exploring the hypothesis of the pathway of MT in high-grade astrocytoma (HGA). The present study aimed to compare the molecular alterations between DA and HGA in the same individual using the next-generation sequencing (NGS).

## Materials and Methods

### Study Population

Patients who were diagnosed with DA and HGA in the same patient at different times between 2016 and 2020 were included. The study was performed with the approval of the Human Research Ethics Committee of Faculty of Medicine, XXX (REC. 61-372-10-1). The informed consents were obtained and signed routinely from all patients before tumor specimen collection at the biobank of the Faculty of Medicine, Prince of Songkla University.

### Frozen Tissue Specimens

Tumor specimens were collected at the time of craniotomy operation and frozen at −40°C until use. Samples were histologically reviewed by a pathologist to confirm the diagnosis at each stage of MT. Using a High Pure PCR Template Preparation Kit (Roche, Berlin, Germany), deoxyribonucleic acid (DNA) was extracted from the frozen tumor specimens. DNA extraction was performed according to the manufacturer's instructions as per the previous study. 7 8

### Next-Generation Sequencing and Data Processing

Following basic quality control assessment, the DNA libraries were sequenced on an Illumina NovaSeq 6000 platform with 150 pair-end read format with an average depth of 40 *x* (median = 30 *x* ). The raw sequencing reads were achieved using FastQC; therefore, trimming and mapping to the human genome (GRCH38, UCSC hg38) using the Burrows-Wheeler Aligner (BWA) software program. 9 Using the Genome Analysis Toolkit (GATK), the indel realignment over the overlapping target regions was performed. 10 Hence, SnpEff was used to do the variant calling and identify single nucleotide polymorphism (SNP), and insertion and deletion were annotated.

### Statistical Analysis

The association between gene mutation and clinical data was analyzed using the chi-square test and/or Fisher's exact tests. Hence, the Mann–Whitney's *U* test was used to compare the mean between the two groups. The statistical tests were two sided, and a value of *p* < 0.05 was considered statistically significant. Moreover, clinical characteristics and magnetic resonance imaging (MRI) of the brain were collected from medical records and databases for clinical correlation propose. The waterfall plots were performed to demonstrate mutation landscape using R version 4.0.4 with the “GenVisR” package. 11 12 Moreover, the MRIs of the brain were integrated into the waterfall plots for visualization.

## Results

### Patient Demographics and Clinical Characteristics

Twelve tumor specimens from five patients were included in the present study and the male-to-female ratio was 0.20:1. The median age at diagnosis was 45 years (interquartile range: 10) and all tumors involved the frontal lobe. The mean follow-up duration for the present cohort was 50 months and the baseline clinical characteristics and outcomes are revealed in Table 1 . The extent of resection was that the majorities were partial resection following adjuvant treatment. Twenty-five per cent of the present cohort received temozolomide when DA transformed to glioblastoma because the high cost of temozolomide is the major cause that temozolomide has not been implemented as the standard treatment in health resource-limited settings. Hence, DA patients had a median MT-free time of 7 months (95% confidence interval [CI]: 0.14–14.63) and had a poor prognosis with a median survival time of 17 months (95% CI: 14.85–19.14). Moreover, we then analyzed the associations between the presence of MT and baseline characteristics of patients. As a result, the clinical characteristics did not significantly associate with MT of DA. Following DNA extraction, nine samples passed quality check control with an average quality check control for NGS.

**Table 1:** Demographic data of astrocytoma patients with malignant transformation

Sample	Histopathology	Time to MT, mo	Extent of resection	Adjuvant therapy	Survival time, mo
Patient 1 (48-y-old woman)					
T1	Diffuse astrocytoma at the left frontal lobe		Partial resection (70%)	–	
T2	Anaplastic astrocytoma	1 mo after first operation	Subtotal resection (80%)	RT	
T3	Glioblastoma	4 mo after first operation	Subtotal resection (80%)	Re-RT with TMZ a	Death at 23 mo after first operation
Patient 2 (45-y-old man)					
T4	Diffuse astrocytoma at the left frontal lobe		Subtotal resection (80%)	RT	
T5	Glioblastoma	44 mo after first operation	Total resection	Re-RT with TMZ	Alive (50 mo after first operation)
Patient 3 (28-y-old woman)					
T6	Diffuse astrocytoma at the right frontal lobe		Partial resection (70%)	RT	
T7†	Anaplastic astrocytoma	7 mo after first operation	Subtotal resection (90%)	Adjuvant vincristine/cyclophosphamide	
T8	Glioblastoma	14 mo after first operation	Partial resection (70%)	Best supportive care	Death at 16 mo after first operation
Patient 4 (58-y-old woman)					
T9 b	Diffuse astrocytoma at right parasagittal area		Neuronavigation-guided biopsy	RT	
T10 b	Glioblastoma	15 mo after first operation	Subtotal resection (80%)	Re-RT	Death 17 mo after first operation
Patient 5 (32-y-old woman)					
T11	Diffuse astrocytoma at the right frontal lobe		Partial resection (70%)	−	
T12	Glioblastoma	3 mo after first operation	Partial resection (70%)	–	Death at 5 mo after first operation

Abbreviations: MT, malignant transformation; RT, radiotherapy; TMZ, temozolomide.

a Progression of the residual tumor within 1 month following third operation and two reoperations were performed later (total operations were five).

b Tumor specimens did not pass quality control before whole exome sequencing.

### Mutational Landscape in DA with MT

Genetic alterations which were frequently found among samples were *IDH1* (rs34363027, rs386392441, rs71412484, rs1446325, rs57383668, rs796498057), *IDH2* (rs60147683, rs2970357), *GGT1* (rs768399767), and *KMT2C* (rs58528565), as shown in Table 2 . *IDH1* mutation was present in all specimens, but we did not find the *IDH1* R132H hotspot mutation in our cohort. However, we observed the dose–response relationship of insertion of T-nucleotides and MT of DA. CT/CTT polymorphism was found in the pre-MT stage of DA, while higher poly-T insertion (CTTT/CTTTT) polymorphism was found in HGA. Moreover, HGA had a significantly higher mean number of T insertion than DA (Mann–Whitney's *U* test, *p* = 0.02), while other SNPs did not significantly differ between the two groups using Fisher's exact test.

**Table 2:** Genetic alterations of *IDH1* , *IDH2* , and other essential genes

rs	Gene	Chrom	Ref	Alt	Type	Position	Effect	COSM ID	Sample
rs1446325	*IDH1*	2	C	T	SNP	209120640	Upstream gene variant	–	T01, T02, T03 T04 T06, T08 T11, T12
rs57383668 rs796498057	*IDH1*	2	GA	G	DEL	209101905	Intron variant	–	T01, T02, T03 T04, T05 T06, T08 T11, T12
rs34363027 rs386392441 rs71412484	*IDH1*	2	C	CT\CTT	INS\INS	209110270	Intron variant	–	T01
	*IDH1*	2	C	CTT\CTTT	INS\INS	209110270	Intron variant	–	T02
	*IDH1*	2	C	CTTT\CTTTTT	INS\INS	209110270	Intron variant	–	T03
	*IDH1*	2	C	CTTT	INS	209110270	Intron variant	–	T04
	*IDH1*	2	C	CTTT	INS	209110270	Intron variant	–	T05
	*IDH1*	2	C	CTT	*INS*	*209110270*	Intron variant	–	T06
	*IDH1*	2	C	CT\CTTT	INS\INS	209110270	Intron variant	–	T08
	*IDH1*	2	C	CTT	INS	209110270	Intron variant	–	T11
	*IDH1*	2	C	CTTT	INS	209110270	Intron variant	–	T12
rs11540478	*IDH2*	15	G	A	SNP	90628537	Intron variant	COSM6494434	T01, T02, T03
rs2970357	*IDH2*	15	T	C	SNP	90623052	Intron variant/downstream gene variant	COSM3754569	T01, T02, T03 T04, T05 T06, T08 T11, T12
rs768399767	*GGT1*	22	G	A	SNP	25023537	Missense variant	COSM1032740	T01, T02 T05 T08 T11
rs58528565	*KMT2C*	7	G	C	SNP	151927023	Stop gained	COSM216053\COSM216054	T01, T02, T03 T05 T06 T11

Abbreviations: Alt, alteration; Chrom, chromosome; DEL, deletion; INS, insertion; Type, transcription effect; rs, Ref; SNP, single nucleotide polymorphism.

*IDH2* mutation is one of the genes associated with LGG in the literature review. In the present study, we found the IDH2 R172K hotspot mutation in 33.3% (3/9) of all specimens. *GGT1* and *KMT2C* mutations were frequently found in our cohort at 55.5% (5/9) and 66.6% (6/9), respectively. However, other SNPs did not significantly differ between the two groups using Fisher's exact test.

**Fig. 1:**
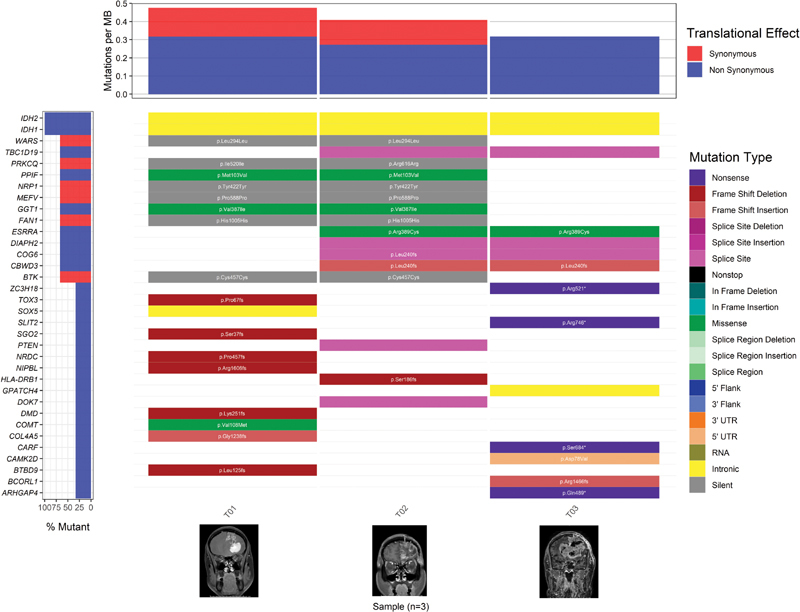
Mutation landscape of malignant transformation in each stage. Waterfall plot shows mutation landscape found of diffuse astrocytoma (T01), anaplastic astrocytoma (T02), and glioblastoma (T03) in the same patient. The type of mutation is represented by different colors. The bottom illustrates contrast-enhanced T1-weighted magnetic resonance imaging of the different stages.

In detail, one patient collected tumor specimens in all stages of MT in the present cohort. *PRKCQ* , *PPIF* , *NRP1* , *MEFV* , *GGT1* , *FAN1* , and *BTK* mutations were found in both DA and anaplastic astrocytoma, whereas mutations of *TBC1D19* , *ESRRA* , *DIAPH2* , *COG6* , and *CBWD3* occurred in HGA. As shown in Fig. 1 , various genetic mutations were found in each stage of MT. Also, *ZC3H18* , *SLIT2 CARF* , *GPATCH4* , *CAMK2D* , *BCORL1* , *ARHGAP4* mutations were found in glioblastoma (T03).

**Fig. 2:**
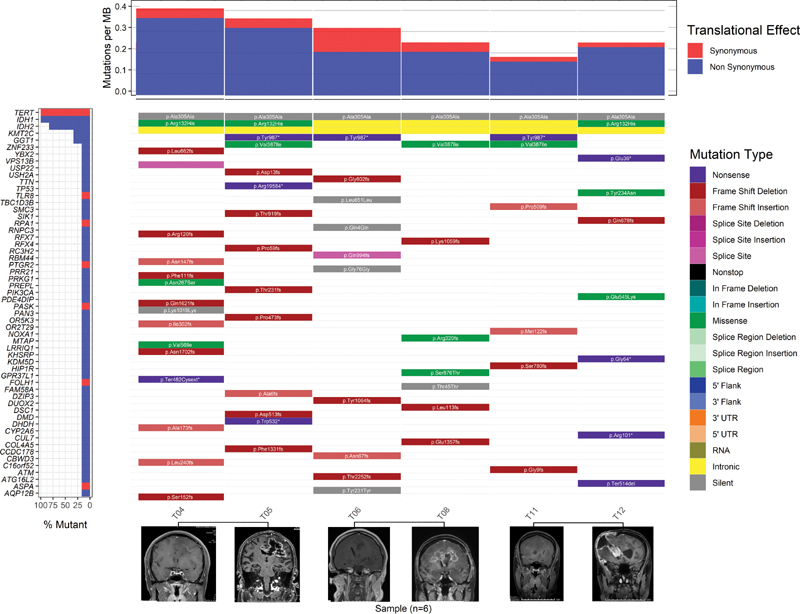
Comparison of mutation landscape between diffuse astrocytoma and glioblastoma. The waterfall plot shows mutation landscape which found diffuse astrocytoma (T04, T06, T12) and glioblastoma (T05, T08, T12) and black solid lines connect the same patient. The type of mutation is represented by different colors. The bottom illustrates contrast-enhanced T1-weighted magnetic resonance imaging of each individual at different stages.

**Fig. 3:**
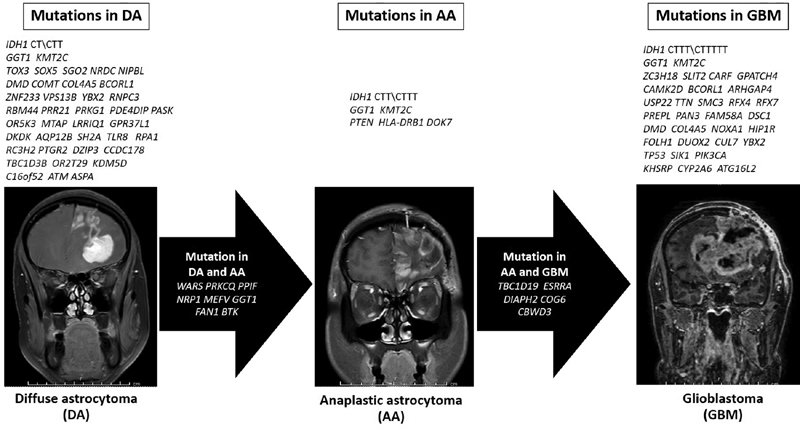
Hypothesized roles of malignant transformation in diffuse astrocytoma.

The remaining patients also compared genetic alterations between DA and glioblastoma, as shown in Fig. 2 . Glioblastoma (T05, T08, and T12) developed mutation as follows: *USP22* , *TTN* , *SMC3* , *RFX4* , *RFX7* , *PREPL* , *PAN3* , *FAM58A* , *DSC1* , *DMD* , *COL4A5* , *NOXA1* , *HIP1R* , *FOLH1* , *DUOX2* , *CUL7* , *YBX2* , *TP53* , *SIK1* , *PIK3CA* , *KHSRP* , *CYP2A6* , and *ATG16L2* . From the present cohort, roles of MT were hypothesized and established for confirmatory study in the future as shown in Fig. 3 . Moreover, we identified other various somatic alterations in each sample based on the Catalogue of Somatic Mutations in Cancer (COSMIC) 13 as a supplement.

## Discussion

MT in DA is an uncommon feature and occurs in approximately 20% of all DA and needs time to develop a malignancy. In the present study, five patients with DA developed MT within 7 months, which was a shorter duration than prior studies that reported the median time of MT was 61 to 68 months in LGG. 1 2 This is potentially explained by the heterogeneity of population studies that the present study focused specifically on DA, while prior studies included DA, oligodendroglioma, and oligoastrocytoma. However, this is in concordance to two previous studies that the prognosis of those with MT is poor. The comparison of genetic mutations among stages of MT may be a way to explore the pathophysiology of MT.

Following the 2016 WHO CNS tumor classification, the mutations of *IDH1* and *IDH2* are the key molecular alterations driving the pathogenesis of gliomas. 14 15 The hotspot mutations of those genes were uncommonly found in the present study, but there was observed evidence of poly-T-nucleotide insertion correlated to MT among DA as the dose–response relationship of poly-T-nucleotide insertion. In detail, more T-nucleotide insertions were significantly observed in higher grade and severity of astrocytoma that may involve splicing errors during transcriptions. This finding is a novel mutation that is found in gliomas, the intronic poly (AT) deletion/insertion polymorphism of the *XPC* gene that has been found in urinary system cancer and breast cancer from literature review. 16 17 Following 32 publications, Dai et al conducted a meta-analysis for identifying the association between this polymorphism and the risk of urinary system cancer in 10,214 cases and 11,302 controls. The results found that polymorphism has a significantly increased risk of urinary cancer. 18 Moreover, this polymorphism has been reported as a risk factor for various cancers such as breast cancer, 17 gastric cancer, 19 and squamous cell carcinoma of the head and neck. 20 21

In addition, we found that *KMT2C* and *GGT1* were common in the present cohort. These molecular alterations have been reported in various cancers, but there was a lack of evidence supporting these mutations in glioma. Cho et al studied the mutation of *KMT2C* in diffuse-type gastric adenocarcinoma and found that these promoted epithelial-to-mesenchymal transition and were associated with a short survival time. 22 Gala et al studied *KMT2C* mutation in breast cancer and found that the deletion of *KMT2C* was associated with poor prognosis via hormone-driven estrogen receptor α activity. 23 *GGT1* transcribes gamma-glutamyltransferase 1 that is associated with a favorable prognosis in renal and ovarian cancers, while these were significantly associated with mortality in the metastatic pancreatic ductal adenocarcinoma. 24 25 However, those need further exploration, the association with MT in DA, from more samples because there was the limitation of a small sample size. The process of MT occurred in the range of 7 to 68 months, and the routine collection of tumor specimens in the tumor bank will increase the number of specimens for further confirmatory study in the future.

Additionally, certain limitations should be acknowledged. The present study explored the genetic profiles at DNA level related to MT; therefore, a lack of comparison between the MT case and the non-MT control groups was observed. Also, studies of the transcriptome and protein expression challenge to confirm these intronic poly-T insertion polymorphisms. However, the present study is the first study to compare molecular profiles in the same patients with different MT stages that establishes the role of MT in DA.

For future research, further studies should be performed on NGS for comparison between MT cases and non-MT controls. Besides, the results of the present study need to be confirmed with molecular findings using more numbers of patients or further study performed in the archival specimens using direct DNA sequencing.

## Conclusion

In summary, we identified a novel significant dose–response association between poly-T insertion polymorphisms that establishes the pathogenesis of MT in DA.
